# Fresh eyes on the European refugee crisis

**DOI:** 10.3402/ejpt.v7.31847

**Published:** 2016-05-12

**Authors:** Eva Alisic, Rianne M. Letschert

**Affiliations:** 1 Monash University Accident Research Centre, Monash University, Melbourne, Australia; 2 Department of Psychosomatics and Psychiatry, University Children’s Hospital Zürich, Zürich, Switzerland; 3 International Victimology Institute Tilburg, Tilburg University, Tilburg, The Netherlands

Over 1 million refugees and migrants have reached Europe via the Mediterranean in 2015. Almost a third of them were children (United Nations High Commissioner for Refugees [UNHCR], 2015a, 2015b). About 1.2 million people started an asylum procedure in European Union (EU) countries last year, more than twice as many as in 2014 (Eurostat, 2016). It is unlikely that the increased influx will soon come to a halt because a peaceful solution to the conflict in Syria—the country of origin of most people who seek asylum—is not in sight (Carter, 2015). The influx involves dire circumstances for many refugees (Turner, 2015), with sad images from Calais in France to Idomeni in Greece, tensions between EU member states and neighboring countries, and social unrest among European communities.

A humane and effective solution is required for what seems an unsolvable problem. In fact, the crisis is a typical “wicked problem” with many facets and complexities. It has health, social, economic, legal, cultural, and logistical aspects, among others. The important issues in the short term (e.g., shelter, safety, and fear within local communities) are distinctively different from those that arise in the long term (e.g., integration, labor and return policies).

Politicians have stated repeatedly that only a collaborative international approach could succeed in addressing these issues (e.g., European Commission, 2015). We believe that the research community has an opportunity^1^ and a responsibility to participate in this effort. As researchers, we can bring together existing insights from different scientific disciplines (see also Schnyder, 2013) and forge new ones in order to improve understanding and increase the potential for creative and innovative approaches. To this end, we organized an interdisciplinary expert meeting, grounded in a collaboration between the Global Young Academy and the Dutch Young Academy.

Young Academies are academic organizations for scientists and scholars, at the beginning of their independent careers, who have been selected for the excellence of their research and a firm commitment to translating academic insights into society (Brück et al., 2010). Membership in a Young Academy is for a limited term, normally 4–5 years. Currently, there are approximately 30 National Young Academies, in addition to the Global Young Academy, providing an opportunity to bring together young experts from a range of disciplines and countries.

With the meeting “Fresh Eyes on the Refugee Crisis,” we intended to put the potential for interdisciplinary contributions to the test. Our specific aims were the following:

1. Develop a brief or statement for national and European policymakers and committed citizens
2. Liaise with international policymakers to ensure the brief is considered
3. Spur an active, nuanced dialog via social and traditional media
4. Encourage interdisciplinary collaboration among excellent young scientists, including scientists who are refugees.

During 2 intensive days in Amsterdam in December 2015, we worked together with 22 experts on various issues relating to migration, refugee flows, and integration of refugees. Participants’ expertise ranged from human rights to history, and from public health to engineering. It was an international team from three continents and also included people from (former) conflict areas. It drew input from a broader online consultation in which we asked young scientists and refugee scholars about theoretical or empirical insights, innovative/unconventional approaches, and unaddressed issues.

## Solidarity

Solidarity emerged as a central theme in the meeting (Participants of Fresh Eyes on the Refugee Crisis, 2016). It is one of the fundamental values mentioned in the EU's Charter (European Union, 2000). However, current practices regarding refugees do not appear to embody this value. Furthermore, several of our participants stated that the current crisis is rather one symbolizing a political debacle in which threats to the underlying value of solidarity are becoming more and more visible than merely or only a refugee crisis.

Labeling newcomers as asylum seekers, migrants, or even refugees does not facilitate the development of a sense of belonging and rather reinforces “otherness,” which may result in a reduction of policy and community support (Olsen, El-Bialy, Mckelvie, Rauman, & Brunger, 2016). Marginalization, hostility, and discrimination are thought to be drivers for further mental health problems and potential radicalization among newcomers (e.g., Ellis et al., 2015). In brief, more solidarity with people who are fleeing their country because of war or human rights violations is needed.

Similarly, solidarity with citizens of host countries also came up as an important aspect to consider; their concerns should be acknowledged. Our report concludes that EU member states have a responsibility to champion solidarity and facilitate inclusion, taking into account societal concerns. Relatively small changes in the refugee integration system can make it more responsive and interactive, to facilitate solidarity on a local level. For example, local councils can ask refugees and local groups to work together on concrete practical issues.

Adam Westbrook, a young videographer, visualized this issue of solidarity:

**Figure:** *Fresh Eyes on the Refugee Crisis* *from* *GYA* *on* *Vimeo*.

## Labor opportunities

A second central topic involved the importance of access to the labor market and the apparent gaps in labor opportunities. Participants concluded that slow access to the labor market has a severe impact on integration opportunities (Davidson & Carr, 2010). Being able to work gives refugees a sense of independence, self-worth, and dignity. In addition, it has economic and public-finance benefits for the host country. Within Europe, conditions for accessing the labor market vary substantially across countries. In some, access to the labor market can be almost immediate, whereas in others it can take months to years (Organisation for Economic Co-operation and Development [OECD], 2015). Even with access to the labor market, refugees are typically overrepresented in low-paid jobs, even though many are highly skilled.^2^ Academic institutes, health and other accreditation boards, and learned organizations can support proper, accelerated accreditation processes of foreign qualifications and the development of a better understanding of the higher education landscape in source countries.

## Addressing the root causes; the elephant in the room

Even though our intention was to focus on integration of refugees, the need to further explore and address the root causes of refugee flows came up repeatedly in our meeting. Politicians face significant challenges in responding to influxes of refugees and their impact on regional, national, and local levels. However, Europe faces a lack of joint engagement and coherence in addressing the root causes, such as conflict, climate change, socioeconomic marginalization, and political instability. Ending violence and improving local conditions are crucial. In addition to addressing current crises, further crises should be prevented. There is a rich but underutilized evidence base from, for example, genocide prevention studies, international relations, economics, public health, climate science and postcolonial history, which could be connected much more. In other words, there is a need and opportunity to understand and address root causes through an interdisciplinary lens.

## Opportunities close to home

The meeting has spurred a number of ideas of what academic communities can do, in addition to conducting relevant interdisciplinary research:

1. Establish mentorship and fellowship programs
  Within our professional communities, we can create opportunities for refugees to participate, exchange knowledge or ideas, and build connections with local colleagues. For example, members of the Dutch Young Academy have decided to act as intermediary and help refugee scholars to get to know Dutch universities from the inside. This can include invitations to research seminars, access to online databases through hospitality agreements, and support with scholarship or job applications. As another example from the Young Academy world, the Young Academy of Scotland has dedicated four seats in their yearly selection of new members to refugee scholars or scholars at risk, with financial support for travel to meetings.
2. Make research findings available
  To enable not only effective policymaking but also effective clinical practice, it is essential that the current evidence base is available to those outside of academia. We can and should make existing and future research findings regarding refugees, migration, and related topics widely available via publications in open access journals, repositories, and/or personal Web sites.
3. Actively reach out to policymakers and the media
  It is worrisome that people who have been exposed to war are often depicted as migrants seeking prosperity, and the public debate regularly appears to be colored more by ideology than by facts. Our professional community has provided high-quality insights regarding the mental health consequences of war and seeking asylum (see Fazel, Reed, Panter-Brick & Stein, 2012; Hall & Olff, 2016; Porter & Haslam, 2005; Schick et al., 2016). There are many opportunities to make these findings better known, to inform the debate and facilitate trauma-informed care for refugees.
4. Drive or facilitate participatory approaches: involve (former) refugees
  In preparation to our meeting, we asked young scientists and refugee scholars for their insights and input. The involvement of refugee scholars and clinicians is important for many issues, for instance in the assessment of best and bad practices relating to labor market opportunities and of cultural differences that might cause tensions within communities. Also with regard to return policies, the perceptions of refugees on how societies confronted with violent conflict should overcome their violent past, which is often labeled under the heading of transitional justice, are of utmost importance.
  The refugee crisis should be addressed with concerted effort. While this may seem an insurmountable task on an individual level, there are several important and straightforward steps we can take to contribute.

*Eva Alisic*
Monash University Accident Research Centre
Monash University
Melbourne, Australia
Email: eva.alisic@monash.edu Department of Psychosomatics and Psychiatry
University Children's Hospital Zürich
Zürich, Switzerland *Rianne M. Letschert*
International Victimology Institute Tilburg
Tilburg University
Tilburg, The Netherlands

## Footnotes

1 As a recent example, the Directorate General for Research and Innovation of the European Commission organised a two-day conference in February 2016 on how European research can support policy makers. See http://www.ec.europa.eu/research/conferences/2016/migration-challenge/index.cfm.

2 In October 2015, The European Commission launched the “science4refugees” initiative. It aims to enable a match-making process between refugees/asylum seekers with a scientific background and scientific institutions.

## About the authors

Eva Alisic is a senior research fellow at Monash University, currently visiting the University Children's Hospital in Zürich. She is co-chair of the Global Young Academy (www.globalyoungacademy.net).

Rianne Letschert is a professor of victimology and international law at Tilburg University and chair of the Dutch Young Academy (www.dejongeakademie.nl/en).
